# The need for dengue and chikungunya awareness among mental health professionals in Europe

**DOI:** 10.3389/fpsyt.2025.1672597

**Published:** 2025-09-22

**Authors:** Guglielmo Lucchese, Angela Stufano

**Affiliations:** ^1^ Department of Neurology, University of Greifswald, Greifswald, Germany; ^2^ Department of Experimental Medicine, University of Salento, College ISUFI, Lecce, Italy; ^3^ Department of Medical and Surgical Sciences, University of Foggia, Foggia, Italy

**Keywords:** cognition, depression, postinfectious, Arbovirus, Togavirus, Flavivirus

## Introduction

The dynamics of dengue (DENV) and chikungunya (CHIKV) virus transmission across Europe are undergoing a significant transformation, spurred by both global warming and the expanding presence of *Aedes albopictus*, commonly known as the Asian tiger mosquito (1). A recent projection by Farooq et al. suggests that, under severe climate trajectories, the potential for outbreaks of these viruses could multiply nearly five times by the 2060s (1). This points toward a possible transition from occasional flare-ups to more entrenched, continuous transmission in certain parts of the European Union. Italy, in particular, stands out in newly released analyses covering the period from 2006 to 2023, marking it as a key European scenario for arboviral disease emergence (2). The research indicates that local outbreaks are closely tied to the introduction timing and geography of infected individuals. Nonetheless, the data confirm that environmental conditions—such as population density, favorable climate, and the pervasive establishment of *Aedes albopictus*—significantly contribute to sustained transmission.

A third retrospective study analyzed chikungunya cases in mainland Europe over 16 years, combining epidemiological data, genomic surveillance, and clinical meta-analyses. From 2007–2022, 4,730 cases were reported across 22 countries, with the UK, France, Germany, and Italy accounting for most (3). The majority were travel-related, originating mainly from India, the Caribbean, and Southeast Asia, though several autochthonous outbreaks occurred in Italy (2007, 2017) and France (2014, 2017). Peaks in incidence aligned with summer travel seasons. Clinical manifestations were dominated by fever (97.6%), joint pain (94.3%), fatigue (63.5%), and rash (52.3%). Genomic analysis revealed two circulating genotypes, with II-ECSA predominant; the adaptive E1 A226V mutation was associated with outbreaks involving Ae. albopictus. While no cases were recorded in 2023, the study emphasizes that climate change, increased global mobility, and vector expansion keep Europe at risk. Indeed, a review just one year old compared southern and northern Europe with regard to climate change and spread of invasive Aedes mosquitoes, particularly Aedes albopictus (4). Climate change could facilitate future dengue (DENV) and chikungunya (CHIKV) outbreaks in northern Europe. Projections suggest that by mid- to late-century, environmental conditions will increasingly support the establishment of Aedes species in northern Europe. Evidence from southern Europe, where Ae. albopictus is already established, shows that local outbreaks tend to follow within a decade of mosquito settlement. Both viruses—though often mild—can lead to severe disease and high public health costs, especially in immunologically naïve populations. Northern Europe is likely to face autochthonous outbreaks in the coming decades, making surveillance, diagnostic readiness, and climate change mitigation critical for prevention.

These studies point to a growing likelihood of recurring DENV and CHIKV events in Europe, propelled by both climatic shifts and increased human mobility. This underscores the urgent need to strengthen early warning systems and monitoring efforts, especially in areas with ecological conditions conducive to virus persistence, even if they haven’t seen previous outbreaks. Once largely confined to tropical zones, DENV and CHIKV have shown clear potential to establish footholds in new, temperate territories like Europe.

## Acute psychiatric manifestation of DENV and CHIKV infection

Psychotic manifestations during or a few days after acute DENV infection have been described both in adult and pediatric patients (5–7). A recent review by D’Souza and D’Silva described depression, anxiety, mania, and psychosis in association with acute or subacute dengue fever and Dinakaran et al. highlighted anxiety and depression as most frequent psychiatric manifestations of the acute infection (8, 9). Similar reports of psychosis and mania during acute CHIKV infection exist (10, 11). These acute psychiatric manifestations of DENV and CHIKV might be due to encephalitis and encephalopathy (7, 12).

## Chronic psychiatric manifestations of DENV and CHIKV infection

The same reviews of observational studies showing psychiatric manifestation of acute dengue fever also describe long-term post-infectious psychiatric sequelae that might constitute an even heavier burden on mental health and are currently under investigated (8, 9, 13).

Specifically, D’Souza and D’Silva indicated as key predictors illness severity, thrombocytopenia, serotypes DENV-2 and DENV-3, and hospitalization-related stress as key predictors of long-term prenatal consequences. Inflammatory cytokines and epigenetic alterations appear to be central to the proposed pathogenesis. They advocate for long-term psychiatric follow-up and integration of mental health screening in dengue management (8). Dinakaran et al. highlighted how various long-term neuropsychiatric manifestations associated with dengue, including anxiety, depression, mania, psychosis, and cognitive deficits may occur independent of encephalopathy. Mechanisms proposed include cytokine-mediated neuroinflammation, blood-brain barrier disruption, and epigenetic changes such as histone deacetylase (HDAC) activation (9).

Cognition appears also to be chronically affected following DENV infection. A large-scale Taiwanese study (n=244,420) confirmed a modest but statistically significant increase in psychiatric disorders among dengue patients. A specific increases were noted for dementia. The risk was particularly pronounced in individuals over 60 and was evident across sexes (14).

Similarly, depression, anxiety, cognitive dysfunction, and even manic episodes have been reported in CHIKV-infected individuals (15). Studies have shown depression in over 44% of infected individuals in Mexico and memory complaints in 70% of elderly patients in Brazil (15).

Evidence from longitudinal studies specifically indicate high risk of psychiatric sequelae even years after acute infection. A prospective cohort study from Ecuador revealed that dengue survivors experience elevated symptoms of depression and stress in the early months post-infection. At three months, 44.4% of dengue cases had elevated DASS-21 depression scores compared to 17.1% of controls. Stress scores were also significantly higher. While most symptoms normalized over 12 months, neurocognitive testing at one year still showed diminished spatial working memory in cases compared to controls (13). Further longitudinal studies suggest depressive symptoms can persist or emerge years after infection, especially in individuals with chronic arthralgia (13). A nationwide longitudinal Taiwanese study analyzing over 45,000 DENV patients found significantly increased risks of depressive disorders across all timeframes post-infection: <3 months (aSHR 1.90), 3–12 months (aSHR 1.68), and >12 months (aSHR 1.14). While overall anxiety risk was not elevated, hospitalized patients showed increased short-term anxiety risk. Sleep disorder risk rose only during the 3–12 month period (16).

Mechanistically, inflammatory cytokines such as IL-6 and TNF-α may be involved in neuropsychiatric effects (17). Routine psychiatric screening in CHIKV patients, especially older adults, are advocated and the lack of standardized management guidelines currently hinders optimal treatment (17). Farias et al. also highlight the psychiatric burden of CHIKV, citing case reports and small studies that document mood disorders like depression, anxiety, and bipolar exacerbations during the chronic phase (17). They again emphasize that persistent inflammatory states, particularly elevated IL-6, may contribute to psychiatric symptoms and call for urgent large-scale, longitudinal research to elucidate mechanisms and risk factors and to develop proper clinical management strategies (17). CHIKV just like DENV also seems to affect long-term cognitive functions. An empirical investigation on cognitive dysfunction post-CHIKV infection in older adults reveal that CHIKV infection is significantly associated with impairments in executive function, memory, and processing speed (18). About 20% of patients also reported depressive symptoms (18). This suggests that CHIKV’s impact on mental health is not just transient but potentially long-lasting and functionally debilitating in older populations. The relevance of the cognitive impact of CHIKV in geriatric populations lies in a potential increase in dementia in a region like Europe, where the prevalence of cognitive impairment is rising because of population aging in spite of a falling incidence (19, 20).

## Discussion

The recent and foreseeable increase in DENV and CHIKV cases in Europe requires better physician awareness and quicker case identification to enhance early detection and mitigate future risks. Among European physicians, psychiatrists especially need to be on the lookout because DENV and CHIKV may induce a wide array of psychiatric manifestations with a large degree of overlap with other secondary and primary mental health disorders. Prompt diagnosis of acute and chronic mental complications of DENV and CHIKV is of paramount importance for a variety of reasons. First, it allows to optimize the treatment of patients with DENV and CHIKV related mental health conditions that otherwise would remain etiologically uncertain. Second, it facilitates posthaste and effective implementation of public health measures. Finally, it permits the accumulation of data and knowledge on the etiopathogenesis and the mechanisms of arbovirus-induced mental health sequelae paving the way for therapeutic developments. Recommended measures for practicing mental health professionals include a thorough medical history including vaccinations, recent and remote clinical signs of infection, as well as travel and occupational history; careful evaluation of hematological, hepatic, renal, and inflammation laboratory parameters; prompt serological testing in suspected cases; and molecular testing in suspected acute cases.
